# Photostability and toxicity of finasteride, diclofenac and naproxen under simulating sunlight exposure: evaluation of the toxicity trend and of the packaging photoprotection

**DOI:** 10.1186/1752-153X-7-181

**Published:** 2013-12-10

**Authors:** Maria Pia Sammartino, Mauro Castrucci, Daniele Ruiu, Giovanni Visco, Luigi Campanella

**Affiliations:** 1 Chemistry Department, “La Sapienza” University, P.le Aldo Moro 5, Rome 00185, Italy

**Keywords:** Photostability, Toxicity, Packaging, Finasteride, Diclofenac, Naproxen

## Abstract

**Background:**

Drugs photostability plays two different opposite roles; a real advantage arises considering the longer expiration time of the drugs while the consequent persistence in the environment involves an obvious negative effect bound to their harmfulness.

On this basis we tested the photostability and toxicity of three pharmaceutical active principles: Finasteride, Diclofenac and Naproxen. The pure active principles, as well as commercial drugs containing them, were considered; for the last, the protective effect of the packaging was also evaluated. Samples were irradiated according to the ICH Guidelines for photostability testing (The International Conference on Harmonisation of Technical Requirements for Registration of Pharmaceuticals for Human Use); a simulating sunlight source (a mercury-vapor lamp coupled to a tungsten filament one) was used to cover the wavelength range 300–2000 nm; Temperature, Relative Humidity, Irradiance and Illuminance were maintained constant during the photodegradation. The concentrations of the pharmaceutical active principles during the photodegradation were monitored by HPLC with UV/Vis detector. Toxicity tests were performed by means of an amperometric biosensor based on suspended yeast cells. Since the products obtained by the photodegradation process can result as toxic or more toxic than the original molecules, tests were performed first and after the photodegadation.

**Results:**

After 90 hours of exposure the concentration resulted lowered by 42.9%, 88.4% and 91% for Finasteride, Naproxen and Diclofenac respectively. Toxicity of the pure active principles follows the same order of the photostability. After photodegradation a contribute of the reaction products was evidenced.

**Conclusions:**

The simple and cheap analytical procedure here proposed, allowed to obtain not only data on photostability and toxicity of the pure active principles but, even if roughly, also useful information on the reactions kinetic and toxicity of the photodegradation products.

## Background

From several years it is well known [1] that many compounds, including drugs, are photoreactive. In the past, the topic received scarce attention due to the incorrect belief that the packaging could protect the active principle so avoiding its photodegradation [2]. This led to a limited knowledge of the phenomenon until relatively recent time. The European Pharmacopoeia [3] states that a drug must be opportunely protected from the exposure to the light in order to avoid a reduction of the active principle concentration with a consequent lost of efficiency. Photodegradation was then recognized as an important limiting factor of the shelf life of a drug that can be greatly influenced by its wrong storage and/or handling. Thus not only quality control laboratories, but also international organization (like WHO) [4] and pharmaceutical companies [5,6] are interested in the development of a valid protection to preserve active principles from possible degradation. Degradation products could be more toxic than the starting reagents causing side effects on humans and producing unknown biological effects and new risks for ecosystems.

With the introduction of ICH guidelines [7] for carrying out photostability tests on drugs, researchers have deepened studies on the topic and several drugs were individually examined [8,9]; on the contrary, comparative studies on different drugs are still scarce. Such comparison can allow to obtain a scale of stability/recalcitrance (environmental persistence) of similar or different pharmaceutical formulations useful for solving ecopharmacology problems [10] (studies of the interaction between the environmental compartments and drugs for human and animals, products for personal care, for hospital cleaning, disinfectants, antibacterials). Here, we present a research aiming to compare the photostability of three active principles chosen among the most consumed in Italy [11]: Naproxen [12,13], Diclofenac [14,15] and Finasteride [16] (Additional file 1: Figure S1). The first two are well-known anti-inflammatory active principles while the third one is an inhibitor of 5-alpha-reductase (it inhibits the reaction of testosterone to dihydrotestosterone) and it is commonly used for the treatment of prostatic hypertrophy and androgenetic alopecia. Photodegradation tests were carried out using an irradiation source simulating sunlight (see Experimental paragraph). The photodegradation products were not determined because of their possible large number; we preferred to correlate their formation during the degradation with the toxicity trend. Really such data can be more useful since they take into account synergic effect of both the residual drug and all the photodegradation products. The active principles were determined by HPLC using UV/Vis detection. Absorption spectra of the pure molecules (Additional file 1: Figure S2) were recorded in order to set the detector for the chromatographic analysis at the most suitable wavelength. Active principles were subjected to photodegradation as pure molecules (mostly dissolved in water), and as solid pharmaceutical forms; in the last case, they were exposed inside the marketind pack as well as inside and outside the immediate pack, in order to evaluate eventual differences in the protection ability. Kinetic parameters for the photodegradation reaction were determined for the pure molecules dissolved in aqueous solution. The Integral Toxicity Index (before and after their exposure to the simulated sunlight) was obtained by means of an amperometric biosensor [17] based on suspended yeast cells.

## Results and discussion

The accuracy of the analytical procedure adopted for the three drugs was evaluated considering as “true value” the concentration of the active principle declared by the producers; good inaccuracy values resulted, i.e. below the legal limits set for the title printed on the package [18]. Data are reported in Table 1, where also the precision of the measures can be evaluated.

**Table 1:** Inaccuracy evaluated for the analysis of the three drugs

**Drug (tablets)**	**Active principle**	**Declared concentration (mg)**	**Found concentration (mg) ± SD**	**Inaccuracy (%)**
Voltaren 50	Diclofenac sodium salt	50	51.8 ± 0.2	+ 3.6
Momendol 220	Naproxen sodium salt	220	225.5 ± 3.4	+ 2.5
Prostide	Finasteride	5	5.16 ± 0.07	+ 3.2

Data come from at least 3 measures.

In order to study the photodegradation kinetic and to optimize the chromatographic parameters we started with the analyses of the pure active principles. The choice of the initial concentration has followed the criterion to fall inside the linearity range of both the spectrophotometric and HPLC methods. On this base about 10^-3^ mol/l aqueous solution were used.

**Figure 1:**
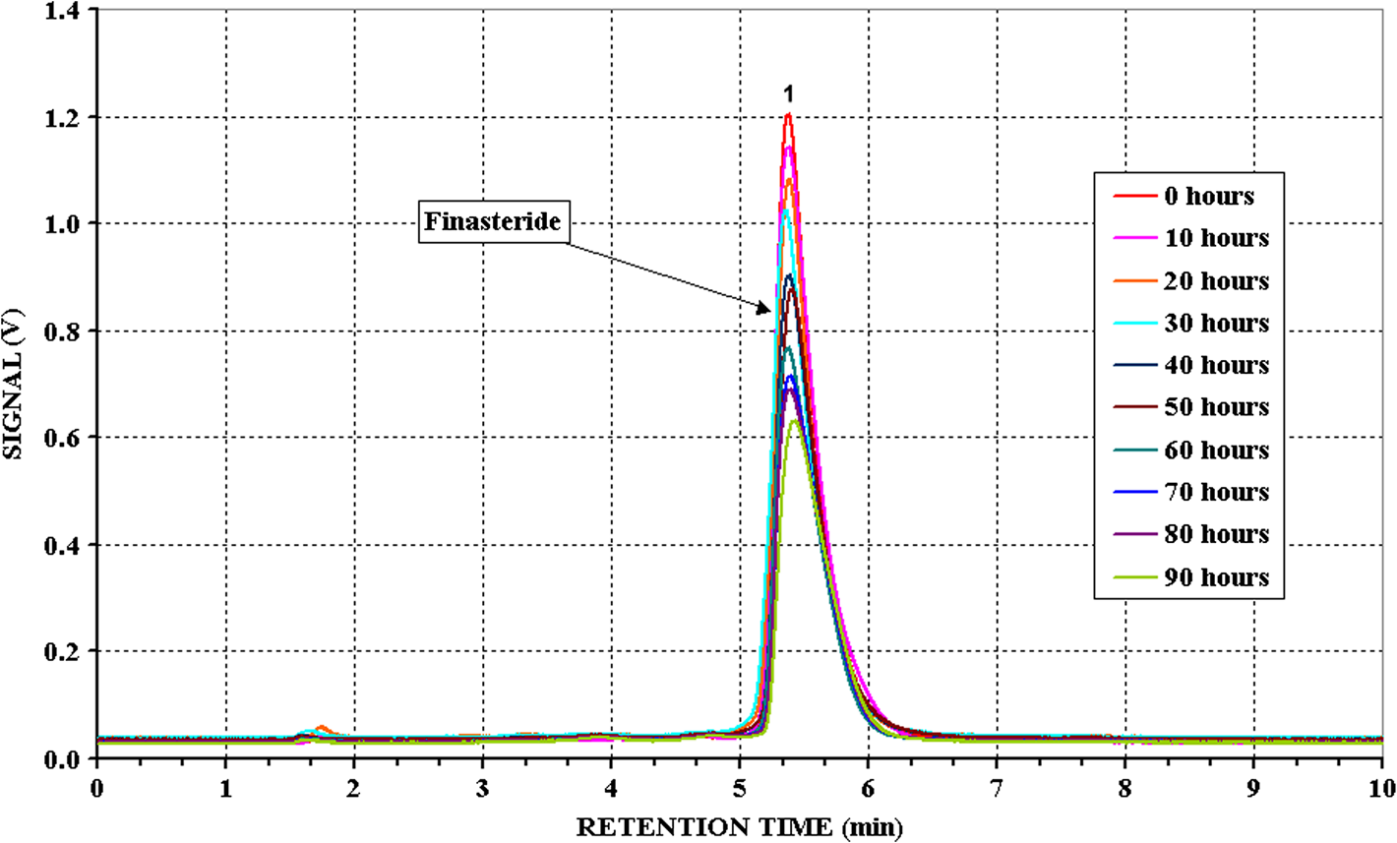
**Chromatograms of Finasteride at different irradiation time.** Initial concentration: 9.992^.^10^-4^ mol/l in 1:20 Acetonitrile/Water solution; eluent: Acetonitrile/Water 95:5; Alltech Alltima C8 5 μm, 250 mm x 4 mm Ø; flow: 1 ml/min.

**Figure 2:**
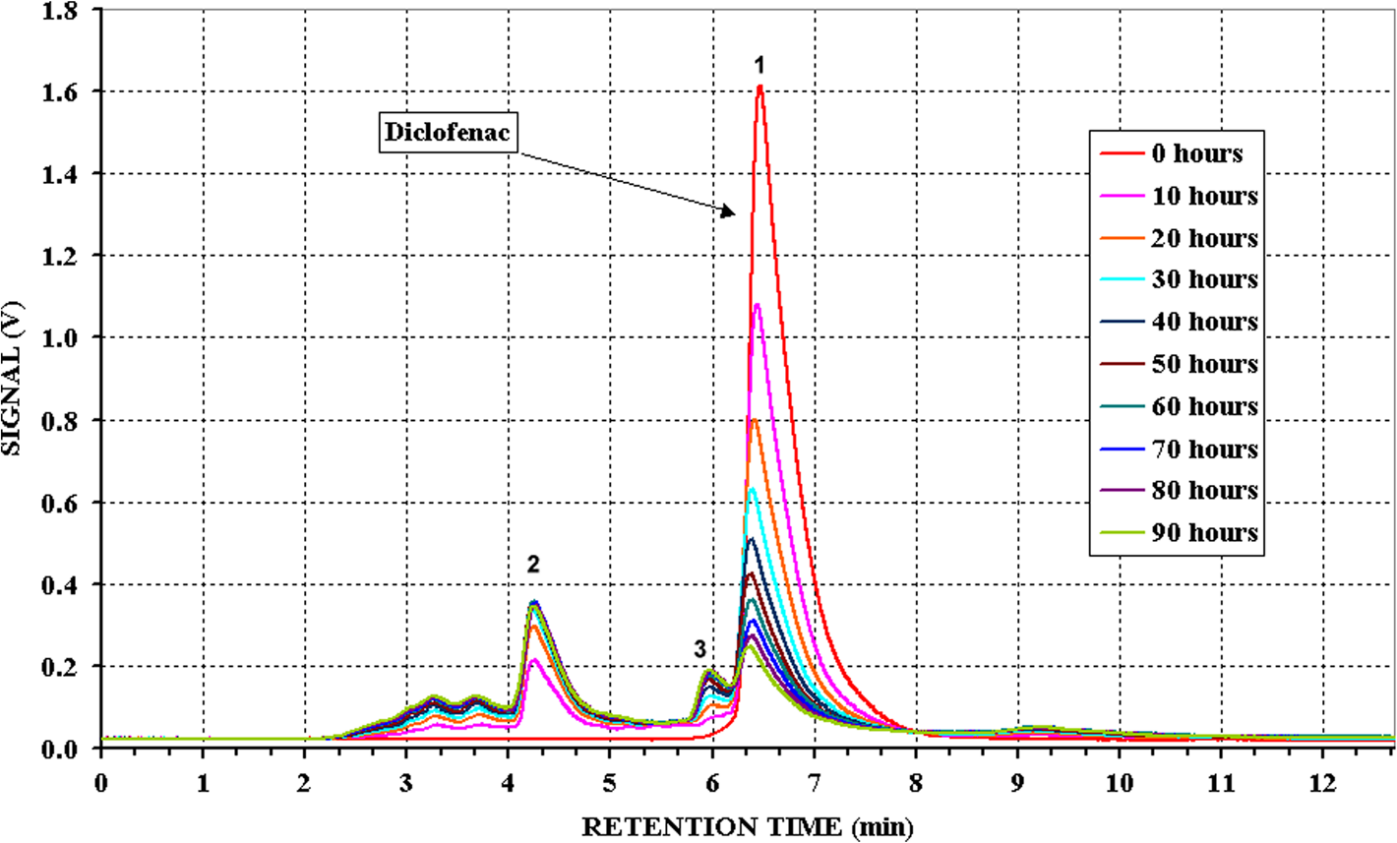
**Chromatograms of Diclofenac at different irradiation time.** Initial concentration: 3.003^.^10^-3^ mol/l in water; eluent: pH 3.3 phosphate buffer/Acetonitrile/Methyl alcohol 30:35:35; Alltech Alltima C8 5 μm, 250 mm x 4 mm Ø; flow: 1.25 ml/min.

**Figure 3:**
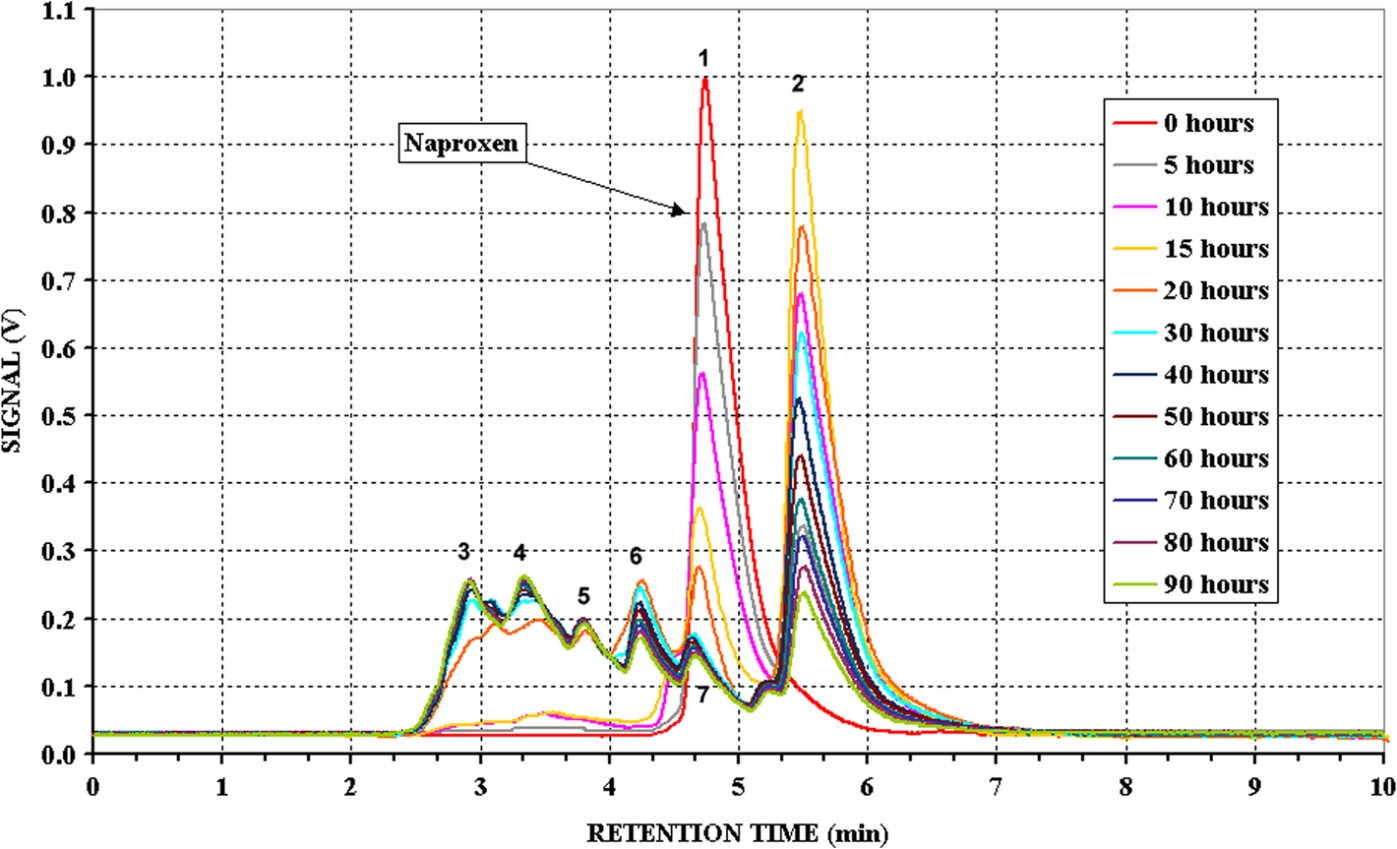
**Chromatograms of Naproxen at different irradiation time.** Initial concentration: 4.893^.^10^-3^ mol/ l in water; eluent: pH 3.3 phosphate buffer/Acetonitrile/Methyl alcohol 30:35:35; Alltech Alltima C8 5 μm, 250 mm x 4 mm Ø; flow: 1 ml/min.

Figures 1, 2 and 3 show the chromatograms obtained for the solutions of the three pure active principles during the photodegradation.

The retention time of Finasteride (Figure 1) is 5′ 25″. The chromatographic peak decreases enough slowly (about 50% at the end of the test) and no other detectable peaks at the fixed wavelength appear. This could means that the basic structure is destroyed during the photodegradation and the formed fragments, with absorption at different wavelength, are less recalcitrant so that they undergo photodegradation preferentially, compared to Finasteride.

In the case of Diclofenac (Figure 2), having a retention time of 6′ 30″, the chromatographic peak decreases enough quickly during the first 30 hours (about 60%) and then the process slows reaching a photodegradation percentage of about 85%. With a similar speed trend, peaks of the reaction products appear in the chromatogram at lower retention times (between 3 and 5 minutes). In particular the peak at 4′ 20″ increases up to reach the maximum after 30 hours of degradation, and then remain constant up the end of the experiment. This could indicates that, after 30 hours, this product also undergoes degradation and concentration remains almost constant as a consequence of a similar speed of formation from Diclofenac and photodegradation. More, the appearance of peaks with absorption at the same wavelength can mean that the basic structure remains intact in the first phase of the process, in agreement with its aromatic nature.

In comparison with the previous two, Naproxen (retention time of 4′ 45″) undergoes a very quick photodegradation (Figure 3); after 30 hours of irradiation its chromatographic peak becomes not detectable. A peak at retention time equal to 5′ 30″ quickly increases reaching its maximum after 15 hours of irradiation and then just as quickly decreases indicating that also its photodegradation starts. Other smaller peaks appears between the 2′ 50″ to 4′ 35″, also attributable to the formation of degradation products, they become significant after 20 hours and remained almost constant up the end of the test. The same considerations made for the Diclofenac may be valid for Naproxen, the largest number of peaks is consistent with its naphthalenic structure.

In Table 2 the retention times of the three active principles and of their photodegradation products are listed.

**Table 2:** **Retention time of the three active principles and their fragments coming from the photodegradation (see Figures**1, 2 and 3)

**Active principle**	**Peak**	**Retention time**
**Finasteride**	1	5′ 25″
**Diclofenac**	1	6′ 30″
Diclofenac fragment	2	4′ 20″
Diclofenac fragment	3	5′ 55″
**Naproxen**	1	4′ 25″
Naproxen fragment	2	5′ 30″
Naproxen fragment	3	2′ 50″
Naproxen fragment	4	3′ 20″
Naproxen fragment	5	3′ 45″
Naproxen fragment	6	4′ 15″
Naproxen fragment	7	4′ 35″

Since we chosen to use a simple and cheap analytical method to follow the photodegradation trend, we are unable to recognize the reaction products and to fully understand the kinetic pattern; anyway, taking into account the formula structures, the noticeable differences in the chromatograms allow us to make some assumptions.

**Figure 4:**
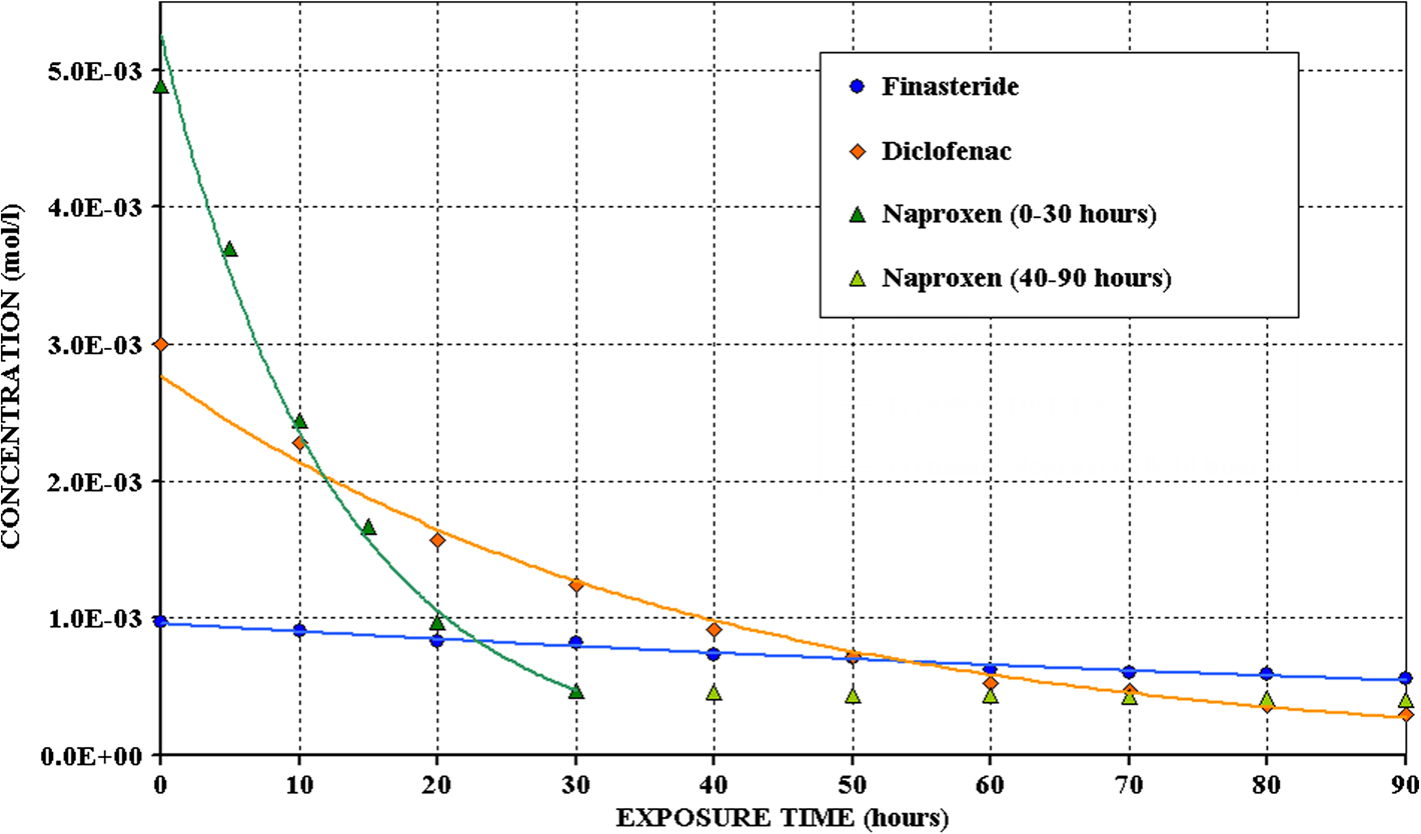
Trend of the Finasteride, Diclofenac and Naproxen concentrations during the photodegradation tests.

Figure 4, where average concentration values vs. exposure time are reported, shows that, for Finasteride and Diclofenac, the trend lines reveal a first order kinetic (C_t_ = C_0_ · e^-kt^) during all the exposition time (R^2^ = 0.9850 for Finasteride and R^2^ = 0.9962 for Diclofenac). The half-life time (t_1/2_ = (ln2)/k) results to be 106.6 and 24.2 hours for Finasteride and for Diclofenac respectively.

Naproxen trend follows the same kinetic equations only during the first 30 hours of irradiation, probably, as above said, this must be ascribed to the simultaneous photodegradation of its degradation product. In the first part, the decay curve fits a first order equation with an enough good correlation coefficient (R^2^ = 0.9855); basing on it, the half-life time results equal to 10.0 hours.

Toxicity tests were carried out, first and after the photodegradation tests, using yeast cell and a Clark oxygen electrode (see Experimental paragraph).

**Figure 5:**
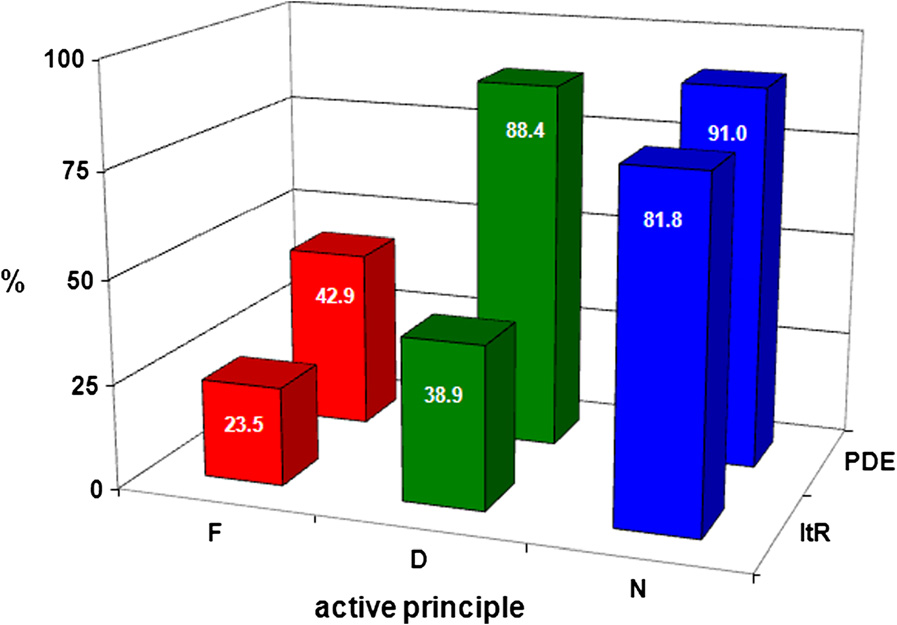
**Comparison between photodegradation efficiency (PDE) and reduction of the toxicity index (ItR) toxicity abatement after 90 hours of exposure to simulated sunlight.** F: Finasteride, D: Diclofenc, N: Naproxen.

Table 3 shows that, for the three pure principles, the initial toxicity follows the order Naproxen = Diclofenac < Finasteride while at the end of the tests the order results D < F < N with a toxicity decrease in all the cases. The toxicity decreases can be better understood basing on Figure 5 where the percentage reduction of the toxicity indexes (ItR) are compared with the photodegradation percentages (PDE). The ItR follows the same trend of the PD but, really, the percentages toxicity reduction always results lower than the photodegradation percentage; this means that surely the photodegradation products are also toxic even if at a lower extent respect to the corresponding drugs.

**Table 3:** Integral toxicity index of active pure principles before and after exposure to 90 h of simulated sunlight

**Active principle**	**Photodegradation time (h)**	**ΔI**_**0**_	**ΔI**	**I**_**t**_ **= ΔI/ΔI**_**0**_
**Finasteride**	0	3.26 ± 0.02	1.65 ± 0.02	0.51
	90	3.46 ± 0.04	1.35 ± 0.03	0.39
**Diclofenac**	0	3.45 ± 0.02	2.66 ± 0.01	0.77
	90	4.77 ± 0.02	2.25 ± 0.02	0.47
**Naproxen**	0	3.06 ± 0.01	2.35 ± 0.02	0.77
	90	4.06 ± 0.03	0.55 ± 0.01	0.14

Values comes from at least 3 tests.

**Figure 6:**
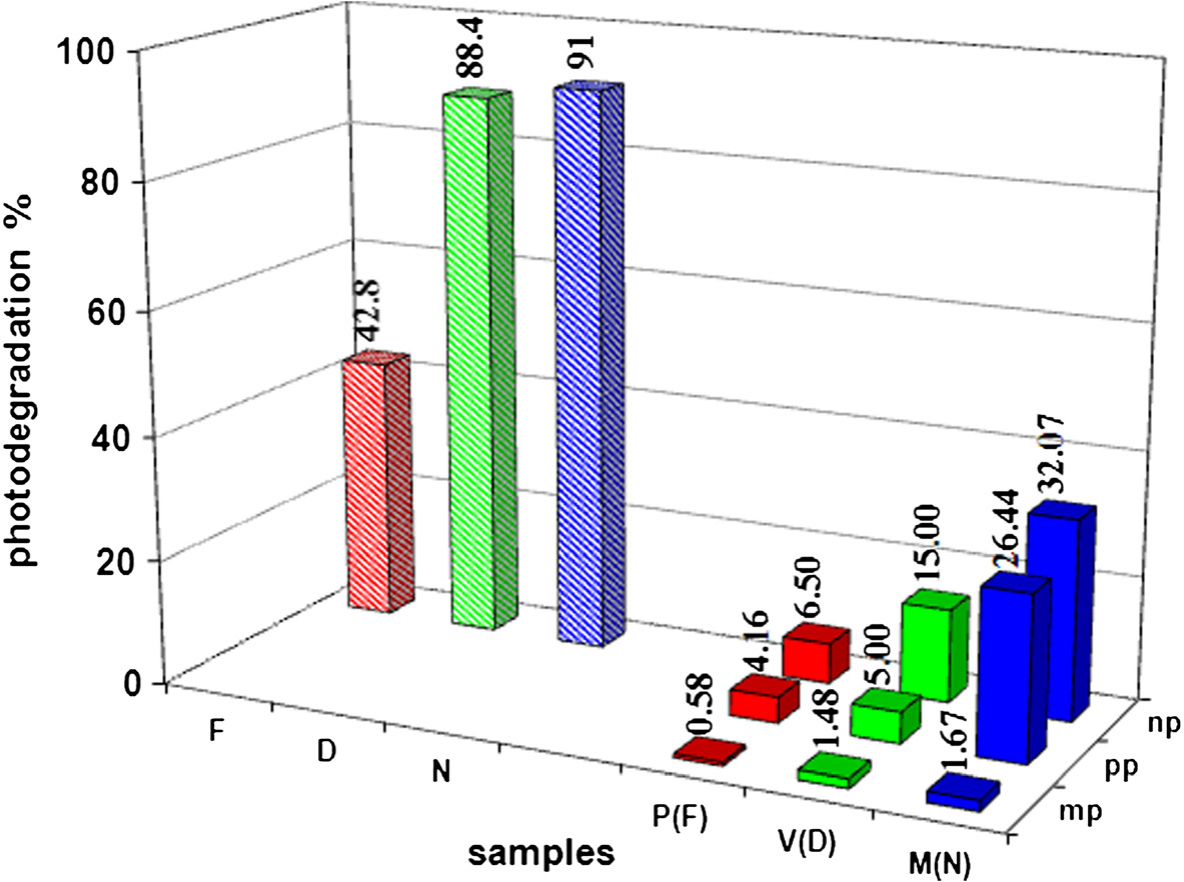
**Photodegradation percentages obtained after 90 h irradiation for both pure active principles and drugs. F**: Finasteride, **D**: Diclofenc, **N**: Naproxen, **P(F)**: Prostide (containing Finasteride), **V(D)**: Voltaren (containing Diclofenac), **M(N)**: Momendol (containing Naproxen); **mp**: marketing package (cardboard), **pp**: primary package (plastic blister), **np**: no package (naked tablet). Data come from at least nine measures, i.e. three measures on each solution obtained from three tests; SD% ≤ 2.5.

Results of the photostability tests are summarized in Figure 6. It can be seen that the photostability order is Finasteride < < Diclofenc < Naproxen for both the pure active principles and drugs containing them. In the last case the difference between Diclofenac and Naproxen is significantly higher with respect to the pure active principles. As expected, their persistence in the environment is significantly lower in aqueous solutions. Surely this is due to a physical protective effect of excipients; as a fact, the single molecules of active principles results differently exposed depending on the different position inside the powder grains and powder layer (even if powders were stratified in a very thin layer as suggested by ICH guideline). Furthermore, other organic compounds contained in the drug can compete with the active principles during the photodegradation. For all the three drugs, the marketing package (cardboard) ensures a good protection against photodegradation while, as expected, the protective efficiency of the primary package is significantly lower. Since we chosen to follow the ICH guidelines, the differences in photostability between the drug naked and inside the primary packaging could be not significant due to the differences of exposed area and thickness.

### Experimental

#### Choice of the light source for the photodegradation

**Figure 7:**
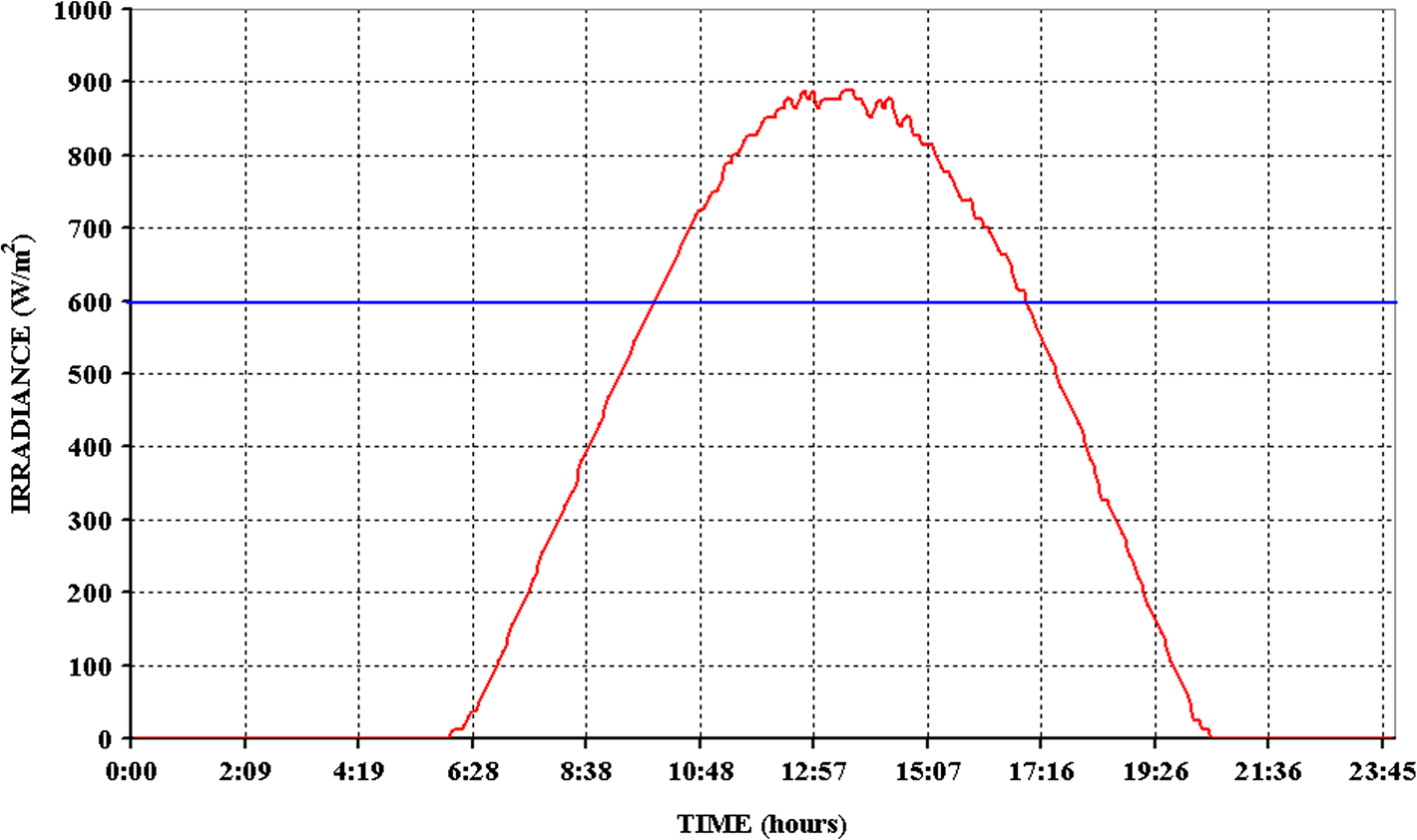
Average solar irradiance measured in Rome (Italy) during a day on July.

Several measures of irradiance and radiant energy were performed on the terrace of the Chemistry Department of Rome “La Sapienza” University; some sunny days were randomly chosen in the months of May, June and July. Relying on these data and complying with ICH guidelines, we have chosen the source to be used for the photostability testing. To evaluate the radiant energy, a polycrystalline photovoltaic panel was connected in series to a 20 Ohm resistance and to a data logger, the voltage was then monitored. The presence of the resistance was needed in order to have the highest possible value of sensitivity of the panel - δV/δ (W/m^2^). It corresponds to a high voltage variation by the slightest variation of radiant energy. A radiometer/luxmeter was placed near to the photovoltaic panel and irradiance (W/m^2^) and illuminance (lux) were measured at about 1 hour intervals from sunrise to sunset. Voltage values, measured at the same times by the photovoltaic panel, were plotted against irradiance measured by the radiometer/luxmeter; a straight line was obtained with a slope of 250 ± 9 W · m^-2^ · V^-1^, an intercept of 6.23 ± 0.06 W · m^-2^ and a good correlation coefficient (R^2^ = 0.9959) (Additional file 1: Figure S3). So, a curve of irradiance was obtained from data collected by the data logger during 24 hours (Figure 7).

Using the PeakFit v4.12 software, the value 2.76^.^10^7^ J/m^2^ was obtained for the area below the curve. It corresponds to the solar energy collected by the photovoltaic panel over 24 hours of an average day (completely sunny) of July and results very close to the one declared on the website of the General Directorate of the European Commission - Joint Research Centre (2.71^.^10^7^ J/m^2^) [19] for a completely July sunny day in Rome. Once established that collected data are significant, the next step was the choice of the irradiance to be used for the tests [20]. Basing on Figure 7, an energy equal to 600 W/m^2^ was chosen that corresponds to the minimum irradiation during the sunniest hours. In such conditions, at least 22 hours of irradiation are needed in order to comply with ICH guidelines (exposure to not less than 1.2^.^10^6^ lux^.^h and 200 W^.^h/m^2^).

Basing on what above said, in order to simulate at the best the solar spectrum, the OSRAM ULTRA-VITALUX [21] Sun Lamp was chosen for the photostability tests. The lamp consists of a tungsten filament (IR-Vis range) and a mercury-vapor lamp (UV range), (Additional file 1: Figure S4). The distance of the source from the sample was optimized in order to provide a value of 600 W/m^2^ that was measured at each sampling time in order to control the lamp’s stability. Eventual sample degradation, due to heating caused by the lamp, was minimized by means of a cooling system providing a laminar air flow. In such conditions, temperature and relative humidity, both monitored by a Lascar Mod. EL-USB 2 data logger, remained almost constant during the tests with values equal to 20 ± 2°C and 42 ± 2% respectively. Experimental conditions adopted for the photostability tests are reported in Additional file 1: Table S1 of the EDF.

#### Integral toxicity test

An already widely applied method [22,23] was used in order to determine the integral toxicity of both the considered active principles, and drugs containing them, first and after the photodegradation tests. The respiratory activity of *Saccharomyces Cerevisiae* suspended yeast cells was evaluated by a Clark oxygen sensor in presence and absence of the sample, an integral toxicity index was then calculated taking into account the difference.

**Figure 8:**
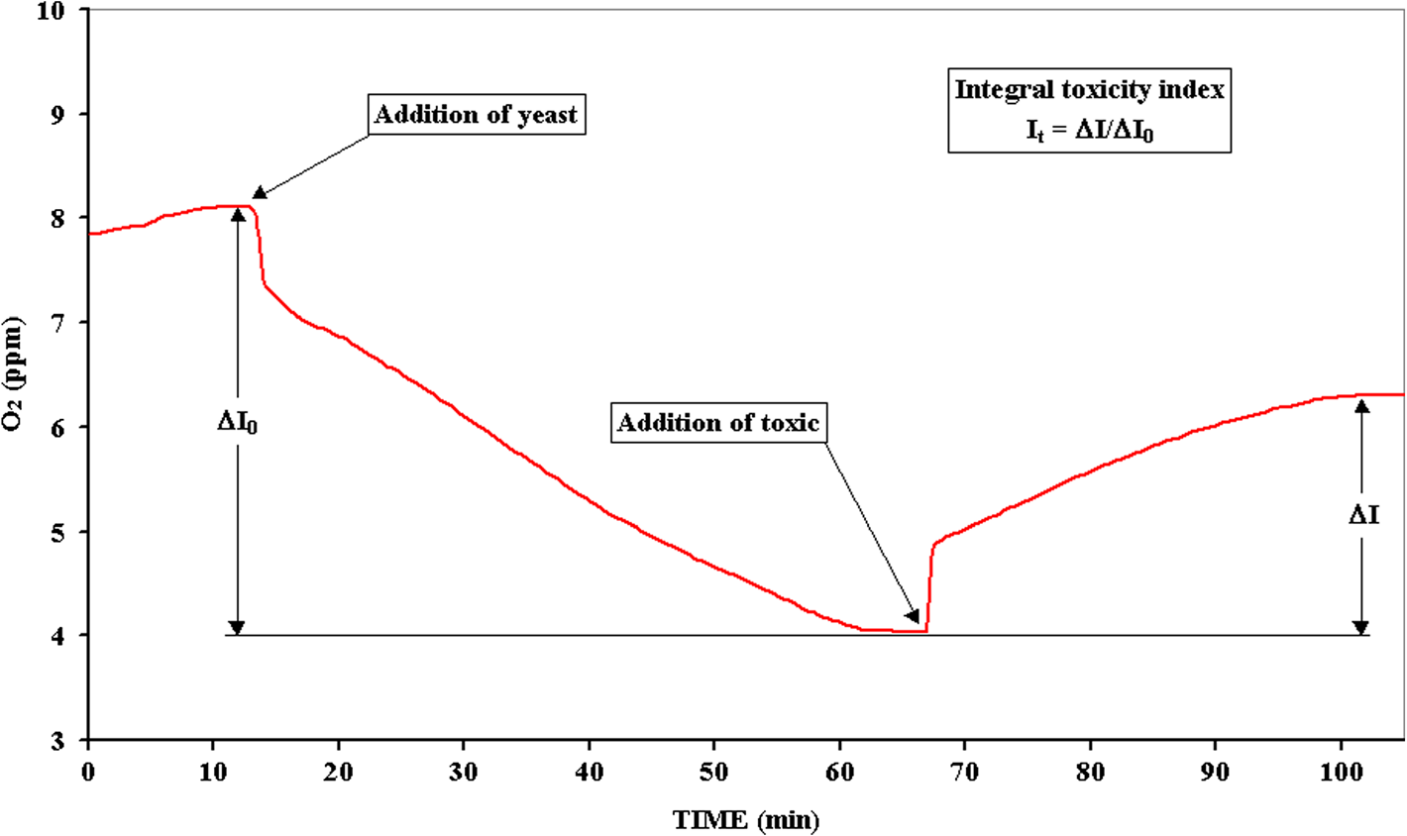
Trends of the oxygen concentration during the toxicity test.

Shortly, 250 mg of yeast cells are suspended in 100 ml of a 1 mol/l glucose aqueous solution (yeast nutrient), for at least 2 hours, to optimize their respiratory activity. A Clark oxygen electrode is then immerged in 10 ml of the previous solution in a thermostated at 25 ± 1°C cell and maintained under magnetic stirring till the stabilization of the signal (usually between 7 and 8 ppm). 1 ml of the sample solution is then added; a decrease of the respiratory function of the yeast, evidenced by an oxygen concentration increase until a new steady-state, is observed that results proportional to the integral toxicity of the sample. The integral toxicity index is calculated as **I**_**t **_**= ΔI / ΔI**_**o**_ (see Figure 8). **ΔI**_**o**_ is then due to the addition of the yeast so being proportional to both the total number of the yeast cells and their breathing activity while **ΔI** is bound to the toxic activity of the sample (concentration and intrinsic toxicity) but also to the number and viability of yeast cells; so, the most accurate toxicity index results to be **ΔI/ΔI**_**o**_.

#### Photodegradation process

Photostability tests were initially carried out on aqueous solutions of the three pure active principles in order to speed up the process and to obtain kinetic information; furthermore, in such conditions we can also have a rough evaluation of their persistence in natural water bodies where they can enter (really at concentration of several order lower than those we here tested) [10]. Solutions were irradiated inside conical flasks of Pyrex England glass because its low absorption at wavelength higher than 320 nm (Additional file 1: Figure S5) complies with ICH guidelines for photostability testing. Irradiation was carried out for 90 hours and, at preset intervals, 0.5 ml aliquots were sampled and stored in the dark until the analysis.

20 tablets of each drug were used for the photostability test: 5 tablets were not radiated in order to use them as reference (blank), while 5 tablets were exposed inside the marketing pack (cardboard box), 5 tablets were exposed inside the primary pack, 5 tablets were exposed by removing all packaging, after a fine grinding and thin layering on glass plate.

#### Calculations for photostability and toxicity test

The photodegradation efficiency (PDE) was calculated as follows

$$\mathrm{PDE}\;=\;\frac{A_{f}}{A_{i}}\times 100$$

where A_f_ and A_i_ are the final and initial areas of the chromatographic peaks registered for the active principle.

Likewise the toxicity reduction (I_t_R) was calculated as follows

$$I_{t}R\;=\;\frac{I_{\mathrm{tf}}}{I_{\mathrm{ti}}}\times 100$$

where I_tf_ and I_ti_ are the final and initial toxicity indexes, calculated as above said.

## Conclusions

Even if simple and cheap, the proposed procedure can be a starting point to obtain useful information about the fate of drugs entering the environment as a consequence of the normal metabolic processes or bad wasting and so on; a more complex procedure (analyte enrichment ) is needed to take into account the very low concentration up today found there [10]. On the contrary, the proposed procedure can be of help in optimizing the process conditions adopted in water treatment plants of pharmaceutical industries; in such case, the concentration is enough high and its decrease need to be speeded up using, as an example, heterogeneous photocatalysis through UV irradiation catalyzed by TiO_2_ that can be combined with visible and/or micro-waves irradiation [24,25]. Also in these cases the coupling of the two analytical techniques, HPLC and toxicity tests, allows us to achieve the goal of obtaining data not only on the photostability but also on non-specific toxicity of the active ingredients and, though roughly, of the photodegradation products. A nonspecific toxicity test is surely more useful, at least for a first approach, than specific ones; in addition, the use of yeast cells respects ethical principles (no use of higher animals) and, especially, the toxic effects on these cells result very similar to those on human ones [26]. The three considered pure active principles, in aqueous solutions, undergo to an enough quick photodegradation with estimated half-life times equal to about 10, 30 and 100 h for Naproxen, Diclofenac and Finasteride respectively. Regarding the drugs, considered that following the ICH guidelines, we posed in extreme conditions, data reveal that the excipients exert a high protective effect against the degradation of the active principle. Surely the marketing package ensure an almost complete protection while, for tests performed on the drug inside the primary pack, we cannot separate the contribute of the excipients from that of the blister but, anyway, especially for Naproxen, photodegradation occurs at a significant level. Such data can be surely useful to establish the drug’s “expiration date” but, as above said, our main goal was to also obtain toxicity data first and after photodegradation. The toxicity order for the three pure active principles results Diclofenac = Naproxen < Finasteride luckily inverse with respect to their photostability. For all the three considered drugs, the percentage toxicity reduction (ItR%) after the photodegradation resulted, at different extent, lower than the photodegradation percentage (PD%) so revealing a toxicity of the photodegradation products. In particular looking at the Figure 5, it could be stated that the toxicity of products coming from the photodegradation of Naproxen are less toxic than those from Finasteride that, in turn, are less toxic than those of Diclofenac but, really such order is not reliable since their different photostability surely plays a role in their toxicity.

What above said highlights the need for further research to obtain complete information, i.e., the use of more advanced analytical techniques, a more detailed time trend for the toxicity and so on.

Surely the topic is of great environmental interest because the drugs are pollutants that tend to increase in concentration, due to both the aging of the population and increase of industrial pollutants [27-29]. Their photostability plays an important role in contrasting their own pollution; as a fact, a low photostability let to a low environmental persistence but a complete toxicity abatement can be attained only after their mineralization [30]. In our tests, may be due to their not adequate irradiation time, the solar light resulted not completely suitable can be so useful in all the cases of unintentional leakage of drugs while inside water treatment plants the process is controlled and speeded up using, as an example, heterogeneous photocatalysis, by TiO_2_, combined with UV and micro-waves irradiation [24,25]. Also in these cases the proposed procedure can be of help in optimizing the process conditions.

## Methods

### Chemicals and drugs

Acetonitrile (99.9%), water plus (distilled water filtered at 2 μm) and methyl alcohol (99.9%), all of HPLC grade, monobasic potassium phosphate (99.0%), orthophosphoric acid (98%) from Carlo Erba Reagenti (Milan, Italy).

Finasteride (N-tert-Butyl-3-oxo-4-aza-5α-androst-1-en-17β-carboxamide; ≥98%), Diclofenac Sodium Salt (2-[(2,6-Dichlorophenyl)amino]benzeneacetic acid sodium salt; ≥98%), Naproxen Sodium Salt ((S)-6-Methoxy-α-methyl-2-naphthaleneacetic acid sodium salt; ≥98%) from Sigma-Aldrich (Milan, Italy).

Momendol 220 tablets containing 220 mg of Naproxen Sodium Salt (Aziende Chimiche Riunite SpA Francesco Angelini, Italy), Prostide, tablets containing 5 mg of Finasteride (Sigma-Tau Industrie Farmaceutiche Riunite SpA, Italy) and Voltaren 50, tablets containing 50 mg of Diclofenac Sodium Salt (Novartis Farma SpA, Italy) were purchased from several pharmacies in Rome, Italy.

All the three drugs are sold in cardboard boxes. The immediate pack of Prostide and Momendol consists of opaque white plastic, while the one of Voltaren consists of transparent colourless plastic. All tablets are covered by a polysaccharide film. The complete compositions are listed in Additional file 1: Table S2 of the EDF.

### Sample pre-treatment

All pure active principles were weighed by means of an analytical balance (0.01 mg resolution) and solutions were prepared using glass flasks of Class A grade. Diclofenac and Naproxen were dissolved in ultrapure water, while for Finasteride a 1:20 CH_3_CN/H_2_O mixture was used because not enough soluble in water.

Tablets of drugs were finely ground in a porcelain mortar and quantitatively racked in a centrifuge tube together with about 6 ml of solvent; the centrifuge tubes were sonicated for 5 minutes and then centrifuged at 10,000 rpm for 10 minutes in order to extract the active principle; supernatants collected from three successive extractions were brought to volume in a suitable flask and analysed by HPLC.

### Instruments

The HPLC equipment consists of a Kontron pump mod. 422, with head pump mod. 420, a Rheodyne valve injection mod. 7125, a 20 μl loop, a Kontron UV/VIS detector mod. 430 connected to a data logger (Pico Technology - Mod. Dr. Daq), a PC equipped with the softwares PicoLog v5.16.2 and PeakFit v4.12. An Alltech reversed phase column (mod. Alltima C8 250 x 4.6 mm I.D.) filled with particles of 5 μm of diameter was used as stationary phase while a mixture of Acetonitrile/Water 95:5 was used as eluent for Finasteride and a mixture of pH 3.3 phosphate buffer/Acetonitrile/Methyl alcohol 30:35:35 for Diclofenac and Naproxen.

A Perkin-Elmer mod. Lambda 16 spectrophotometer was used to select the wavelength to be set for the HPLC detector.

Temperature was maintained at 25 ± 1°C using a Alltech 330 Column Heater thermostatic bath in which the column was immersed.

A radiometer/luxmeter Gossen - model Mavolux Digital was used for the measures of irradiance, illuminance and radiant energy while temperature and humidity were monitored by a data logger Lascar - model EL-USB 3.

The equipment for the Toxicity test consists of a glass thermostated cell; a Clark oxygen electrode Orion 97–08 (Thermo Electron Corporation); an Orion Microprocessor Ionalyzer/901 potentiometer; an Amel Model 868 recorder. *Saccharomyces cerevisie* yeast cells were used as biological mediator.

### HPLC analysis

The wavelength for the HPLC’s UV detector was chosen basing on the UV/Vis absorption spectra of the pure active principles (Additional file 1: Figure S2): 205 nm for Finasteride, 275 nm for Diclofenac and 262 nm for Naproxen. Spectra were acquired in the suitable solvents i.e. the one used as mobile phases for the HPLC analyses. Calibration curves were built not only to follow the photodegradation, but also to test the linear range for each active principle; basing on their lower value, we choose the initial concentration of the pure active principles to be subjected to photodegradation; the criterion was that, after a 90% of photodegradation, the residual concentration had to be appreciated. Additional file 1: Table S3 of EDF summarizes the experimental condition adopted for the analysis as well as the main analytical parameters of the calibration curves obtained for the three active principles.

## Competing interests

Each single author of this paper declares to not have any competing interests of some sort with brands and companies cited in the text. Further, authors are/were not employed in any of the cited company, they have not received fees for consulting or research funding from cited brands.

## Authors’ contributions

LC and GV conceived the research and followed its steps. DR carried out the experimental work and draw preliminary figures and tables summarizing the data; SMP supervised the processing of data checking their analytical consistency and the real utility to achieve the goal of the research. MC along with MPS have been involved in drafting the manuscript and revising it critically for important intellectual content. All authors have read and approved the final manuscript.

## Supplementary Material

Additional file 1: Figure S1 Formulas of the three considered active principles. **Figure S2**. UV/Vis spectra of the three considered active principles. The wavelengths chosen to set the detector are evidenced. **Figure S3**. Correlation between values of tension generated by photovoltaic panel and those ones of radiance measured by radiometer/luxmeter. **Figure S4**. Emission spectra of the Osram Ultra-Vitalux lamp. **Figure S5**. UV/Vis spectrum of the glass constituting the flask used for the photodegradation tests on pure active principles. **Table S1**. Experimental conditions adopted for the photostability test. **Table S2**. Composition of the three considered drugs. **Table S3**. Experimental conditions adopted for the HPLC analysis of the active principles and drugs solution and relative parameters obtained for the calibration curves. Click here for file
