# Supplementary material for: Photostability and toxicity of finasteride, diclofenac and naproxen under simulating sunlight exposure: evaluation of the toxicity trend and of the packaging photoprotection
Source: Chem Cent J. 2013 Dec 10;7:181. doi: 10.1186/1752-153X-7-181 (PMC3881013; doi:10.1186/1752-153X-7-181)
Supplement: Additional file 1: Figure S1 — Formulas of the three considered active principles. Figure S2. UV/Vis spectra of the three considered active principles. The wavelengths chosen to set the detector are evidenced. Figure S3. Correlation between values of tension generated by photovoltaic panel and those ones of radiance measured by radiometer/luxmeter. Figure S4. Emission spectra of the Osram Ultra-Vitalux lamp. Figure S5. UV/Vis spectrum of the glass constituting the flask used for the photodegradation tests on pure active principles. Table S1. Experimental conditions adopted for the photostability test. Table S2. Composition of the three considered drugs. Table S3. Experimental conditions adopted for the HPLC analysis of the active principles and drugs solution and relative parameters obtained for the calibration curves. [file 1752-153X-7-181-S1.doc]

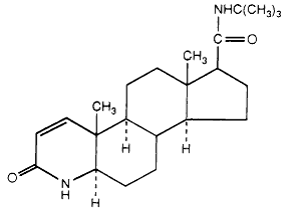


## Finasteride

##
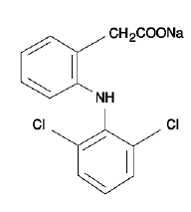

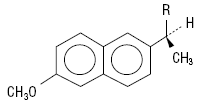
Diclofenac

**Naproxen**

**Fig. A** – Formulas of the three considered active principles.


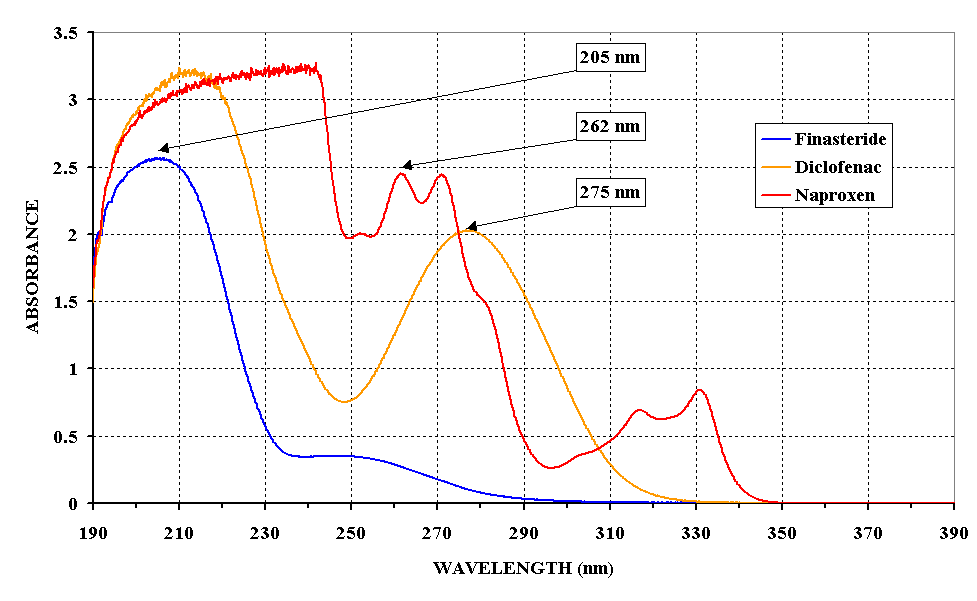


**Fig. B –** UV/Vis spectra of the three considered active principles. The wavelengths chosen to set the detector are evidenced.

**
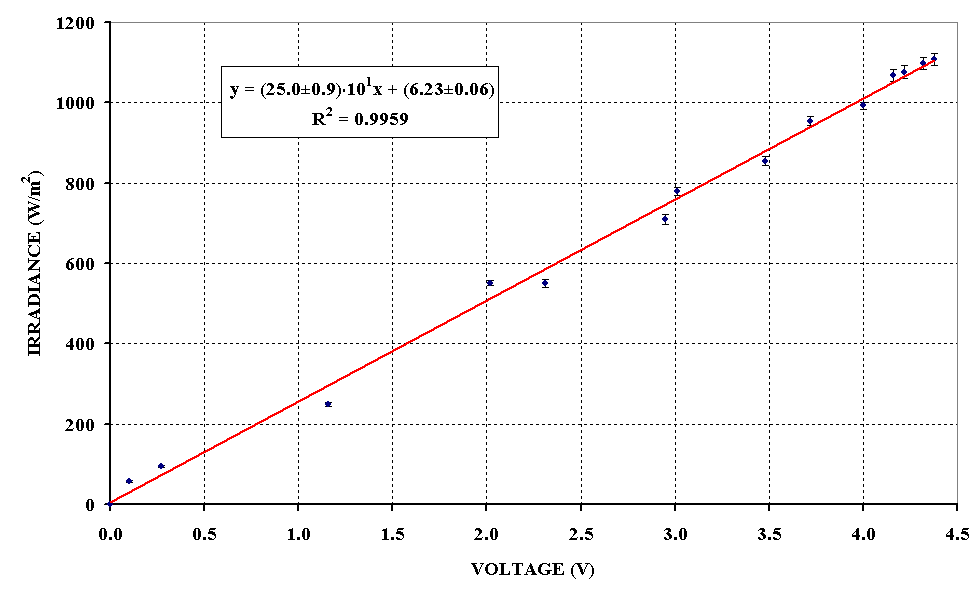
**

**Fig. C** - Correlation between values of tension generated by photovoltaic panel and those ones of radiance measured by radiometer/luxmeter.


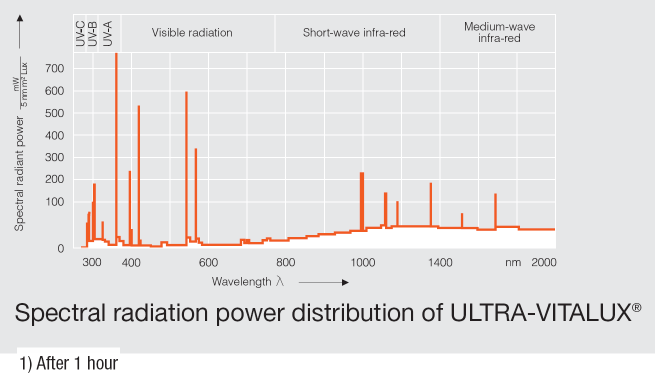


## Fig. D – Emission spectra of the Osram Ultra-Vitalux lamp.


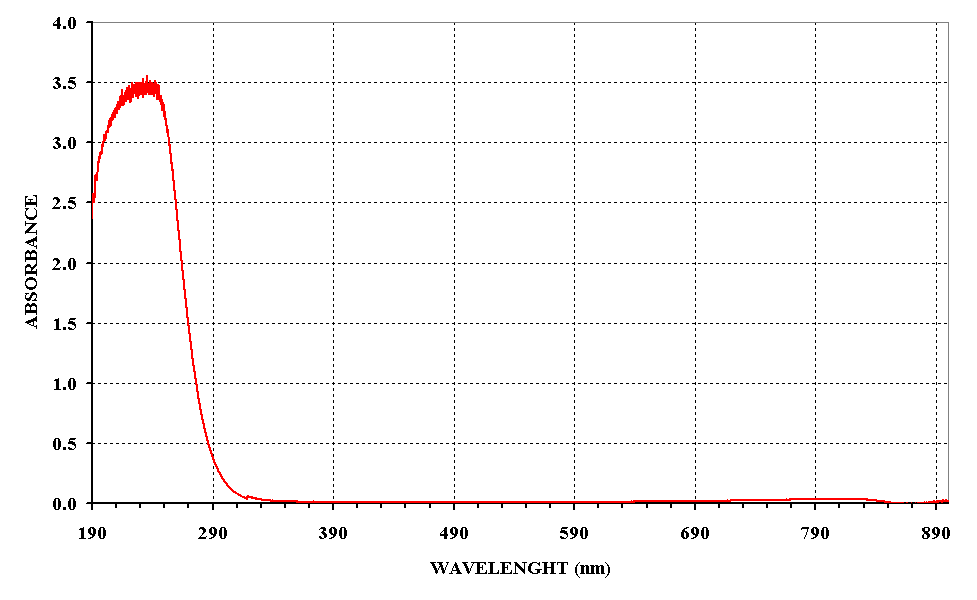


## Fig. E – UV/Vis spectrum of the glass constituting the flask used for the photodegradation tests on pure active principles.

**Tab A –** Experimental conditions adopted for the photostability test

| Temperature (°C) | 20±2 |
| --- | --- |
| relative humidity (%) | 42±2 |
| irradiance (W/m^2^) | 600 |
| illuminance (Klx) | 55 |
| Irradiation time (h) | 90 |

**Tab B** – Composition of the three considered drugs

| **drug** | **compound** | **content (mg)** |
| --- | --- | --- |
| Momendol^1^ | Pharmaceutical form | tablet |
|  | immediate packaging | plastic blister |
|  | external packaging | cardboard |
|  | Naproxen sodium salt | 220 |
|  | Excipients*: | - |
|  | lactose monohydrate | 44 |
|  | corn starch | 28 |
|  | povidone (K25) | 18 |
|  | magnesium stearate | 7 |
|  | microcrystalline cellulose | 21 |
|  | sodium carboxymethyl starch | 10 |
|  | colloidal anhydrous silica | 6.5 |
|  | hypromellose (hydroxypropylcellulose) | 4.5 |
|  | polyethylene glycol 400 (macrogol 400) | 0.9 |
|  | titanium dioxide (E 171) | 0.9 |
|  | talc | 0.7 |
| **Voltaren**^2^ | Pharmaceutical form | tablet |
|  | immediate packaging | plastic blister |
|  | external packaging | cardboard |
|  | Diclofenac sodium salt | 50 |
|  | Excipients*: | - |
|  | corn starch | not declared |
|  | magnesium stearate | not declared |
|  | colloidal anhydrous silica | not declared |
|  | lactose monohydrate | not declared |
|  | microcrystalline cellulose | not declared |
|  | povidone (K25) | not declared |
|  | sodium carboxymethyl starch A | not declared |
|  | talc | not declared |
|  | hypromellose (hydroxypropylcellulose) | not declared |
|  | hydrogenated castor oil | not declared |
|  | red iron oxide | not declared |
|  | yellow red oxide | not declared |
|  | titanium dioxide (E 171) | not declared |
|  | polyacrylate copolymer dispersion 30 per cent | not declared |
|  | silicone antifoam | not declared |
| **Prostide**^3^ | Pharmaceutical form | tablet |
|  | immediate packaging | plastic blister |
|  | external packaging | cardboard |
|  | Finasteride | 5 |
|  | Excipients*: | - |
|  | lactose monohydrate | 106.4 |
|  | pregelatinized starch | 15 |
|  | sodium carboxymethyl starch | 7.5 |
|  | iron oxide yellow | 0.025 |
|  | dioctyl sulfosuccinate sodium | 0.375 |
|  | microcrystalline cellulose | 15 |
|  | magnesium stearate | 0.75 |
|  | hypromellose (hydroxypropylcellulose) | 1.15 |
|  | titanium dioxide (E 171) | 1.04 |
|  | talc | 0.417 |
|  | E132 Indigo carmine over hydrated aluminum | 0.08 |

1. [http://www.gazzettaufficiale.biz/atti/2006/20060171/06A06837.htm]

2. drug datasheet [http://www.pharmamedix.com/diclofenac_specialita_voltaren_compresse_foglietto_illustrativo.php]

3. [http://www.gazzettaufficiale.biz/allegati/09A/09A06502000101/]

**Tab C – Experimental conditions adopted for the HPLC analysis of the active principles and drugs solution and relative parameters obtained for the calibration curves**

|  | | **Finasteride** | Diclofenac sodium salt | **Naproxen sodium salt** |
| --- | --- | --- | --- | --- |
| Column | | Alltech Alltima C8 5 μ m 250mm x 4mm Ø | Alltech Alltima C8 5 μ m 250mm x 4mm Ø | Alltech Alltima C8 5 μ m 250mm x 4mm Ø |
| **Mobile phase** | | Acetonitrile/Water Plus - 95:5 | Acetonitrile/Methyl alcohol/Phosphate buffer at pH 3.3 - 35:35:30 | Acetonitrile/Methyl alcohol/Phosphate buffer at pH 3.3 - 35:35:30 |
| **Loop** (μl) | | 20 | 20 | 20 |
| **Flow** (ml/min) | | 1.000 | 1.250 | 1.000 |
| **Detection λ** (nm) | | 205 | 275 | 262 |
| **Column temperature** (°C) | | 25±2 | 25±2 | 25±2 |
| **Calibration**  **parameters** | **Slope**  (a.u.· mol/l) | 26.4±0.8 | 17.1±0.2 | 10.0±0.3 |
|  | **Intercept** (a.u.) | 0.091±0.003 | 1.07±0.03 | 0.171±0.008 |
|  | **r^2^** | 0.9989 | 0.9996 | 0.9998 |
|  | **Linear range** (mol/l) | 5.10^-5^-1.4.10^-3^ | 1.10^-4^-5.10^-3^ | 1.2.10^-4^-1.10^-2^ |
|  | **LOD** (mol/l) | 2.5.10^-5^ | 5.10^-5^ | 6.10^-5^ |
